# Study on the Corrosion Resistance of Graphene Oxide-Based Epoxy Zinc-Rich Coatings

**DOI:** 10.3390/polym13101657

**Published:** 2021-05-19

**Authors:** Yong Tian, Zhenxiao Bi, Gan Cui

**Affiliations:** 1 School of Science, Qingdao University of Technology, Qingdao 266525, China; 20160015@upc.edu.cn; 2 College of Pipeline and Civil Engineering, China University of Petroleum (East China), No. 66, West Changjiang Road, Huangdao District, Qingdao 266580, China; 18954831115@163.com

**Keywords:** sulfonated, multiwall carbon nanotubes, graphene oxide, epoxy zinc-rich coating, corrosion resistance

## Abstract

In order to improve the corrosion resistance of zinc-rich epoxy coatings and reduce the amount of zinc used, first, graphene oxide (GO) was modified by sulfonated multiwall carbon nanotubes (SMWCNTs) to obtain the modified graphene oxide (SM-GO). The samples were characterized by Fourier transform infrared spectroscopy (FT-IR), X-ray diffraction (XRD) and Raman spectroscopy. Then, four kinds of coatings were prepared, namely pure zinc-rich coating (0-ZRC), graphene oxide-based zinc-rich coating (GO-ZRC), sulfonated multiwall carbon nanotube-based zinc-rich coating (SM-ZRC) and SM-GO-based zinc-rich coating (SG-ZRC). The corrosion resistance of the above coatings was studied by open circuit potential (OCP), electrochemical impedance spectroscopy (EIS), a salt spray test, 3D confocal microscope, and electron scanning electron microscope (SEM). The results indicate that GO is successfully non-covalently modified by SMWCNTs, of which the interlayer spacing increases and dispersion is improved. The order of the corrosion resistance is GO-ZRC > SG-ZRC > SM-ZRC > 0-ZRC. The addition of GO, SMWCNTs, and SM-GO increases the shielding effect and increases the electrical connection between Zn particles and metal substrates, which improves the corrosion resistance. However, SMWCNTs and SM-GO also strengthen the galvanic corrosion, which decreases the corrosion resistance to some extent.

## 1. Introduction

In the field operation, coatings are often used to protect metals to reduce the risk of corrosion. Among anticorrosive coatings, the epoxy zinc-rich coating occupies a certain proportion. The protection of zinc-rich epoxy coating for the metal substrate can be divided into two stages. First, a large number of zinc particles in the zinc-rich coating are used as anodes to provide cathodic protection for the metal substrate. Second, the insoluble corrosion products produced by the reaction of zinc particles fill the defects in the coating and act as a physical shield against corrosive substances [1,2,3,4]. If the zinc-rich coating plays a functional role in cathodic protection, it is necessary to establish an effective/active electronic channel between zinc particles and metal substrate. However, the adhesive and the corrosion products of zinc in the coating are often non-conductive, which leads to the emergence of a lot of isolated zinc particles in the coating. Therefore, it is difficult to provide sufficient protection for the metal substrate [5]. The addition of graphene to the coating promotes the formation of the electron conduction path between zinc particles and metal substrate. The content of zinc in isolation is reduced, and the effect of cathodic protection is enhanced. As an impermeable substance, graphene hinders the corrosive substances to pass through. Therefore, the service life of the coating is prolonged [6,7,8,9,10,11,12].

Owing to the high aspect ratio and van der Waals force, graphene needs to be modified to improve its dispersibility in the coating [13]. Oxidizing graphene to graphene oxide (GO) is the simplest modification method [14]. GO has good dispersion in both aqueous solution and polymer matrix. Many experts have tried to modify graphene in other ways to further improve the dispersion of graphene in the coating. Yang et al. modified the graphene sheet (MGP) using one-dimensional multi-walled carbon nanotubes (MWCNTs). The modified product MWCNTs/MGP was dispersed in epoxy resin, and the scanning electron microscope (SEM) result indicated that the long and zigzag MWCNTs could bridge adjacent graphene sheets and inhibit the stacking of graphene [15]. As a result, MWCNTs/MGP had higher solubility and better compatibility than MGP. Hu et al. studied the effect of carbon nanotubes (CNTs) and GO on the anti-corrosion performance of epoxy coatings. First, GO and CNTs were chemically modified by 3-aminophenoxyphthalonitrile to obtain the nitrile functionalized graphene oxides (GO-CN) and carbon nanotubes (CNTs-CN), respectively. Second, GO-CN was further modified using CNTs-CN to obtain GO-CN&CNTs-CN hybrid, which was added to the epoxy coating. Due to the better dispersion and synergistic effect of GO-CN&CNTs, the epoxy coating showed high corrosion resistance [16]. In addition, we show in Table 1 that other modification methods are used to improve the corrosion resistance of the coating.

The above examples prove that the modification of GO with carbon nanotubes is an effective way to improve the dispersion of GO and enhance the corrosion resistance of the coating. Moreover, carbon nanotubes and graphene are conductive materials that enhance the electrical conductivity between zinc powders. The novelty of our paper is to add graphene modified by carbon nanotubes to zinc-rich coatings. Presently, no relevant research has been reported.

However, there are some controversial findings. Turhan et al. prepared the MWCNTs/Mg composites by adding MWCNTs to pure magnesium, and its corrosion resistance was studied. The results showed that the corrosion rate of MWCNTs/Mg composites was much higher than that of pure Mg [22]. Gergely et al. found that MWCNTs had galvanic corrosion in the epoxy zinc-rich coating, which decreased the anti-corrosion ability [23,24]. Therefore, we want to know what effect the graphene modified by carbon nanotubes will have on the anti-corrosion performance of the epoxy zinc-rich coating.

In order to prove our conjecture, in this paper, MWCNTs were sulfonated first to form sulfonated multiwall carbon nanotubes (SMWCNTs). Subsequently, graphene oxide (GO) was modified by SMWCNTs to improve its dispersion in the solvent [25]. For comparison, four kinds of coatings were prepared, namely pure zinc-rich coating (0-ZRC), graphene oxide-based zinc-rich coating (GO-ZRC), sulfonated multiwall carbon nanotube-based zinc-rich coating (SM-ZRC) and SM-GO-based zinc-rich coating (SG-ZRC). Last, the corrosion resistance and anti-corrosion mechanism of the above coatings were studied.

## 2. Experimental

### 2.1. Materials

Graphite powder (325mesh, 99.95%) and MWCNTs (inner diameter: 3–5 nm, outer diameter: 8–15 nm, length about 50 μm) were purchased from Shanghai Aladdin Biochemical Technology Co., Ltd., Shanghai, ChinaPotassium permanganate (KMnO_4_, 99.5%), concentrated sulfuric acid (H_2_SO_4_, 98 wt.%), sodium nitrate (NaNO_3_, analytical purity), hydrogen peroxide (H_2_O_2_), hydrochloric acid (HCl, 36%), concentrated nitric acid (HNO_3_, 70%), acetone (99%) and anhydrous ethanol (75%) were purchased from Sinopharmaceutical Group Chemical Reagent Co., Ltd., Beijing, China. The epoxy zinc-rich coating with 80 wt.% zinc was provided by Shanghai Qimeng Chemical Co., Ltd. (Shanghai, China). EP002 curing agent was provided by Kunshan North Asia Chemical Co., Ltd, Jiangsu, China.

### 2.2. Preparation of SMWCNTs, GO and SM-GO

#### 2.2.1. Preparation of SMWCNTs

A total of 1 g of MWCNTs were added to 200 mL of mixed acid (*V*_H2SO4_/*V*_HNO3_ = 3:1). After the mixed solution was sonicated in a water bath for 4 h at room temperature, 200 mL of deionized water was added. Then, the mixed solution was heated to 100 °C and stirred for 4 h. After standing for 12 h, delamination occurred in the mixed solution. The sediment was washed to remove surface impurities. Finally, SMWCNTs was obtained by drying for 24 h in a vacuum drying box at 80 °C.

#### 2.2.2. Preparation of GO and SM-GO

**Figure 1:**
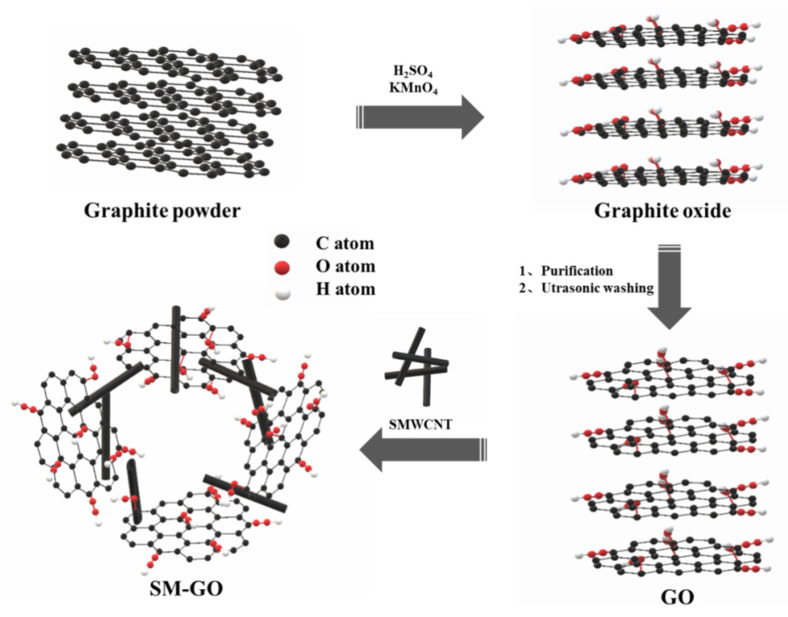
Schematic illustration of the preparation of GO and SM-GO.

GO was prepared by the improved Hummers method. First, 2 g graphite powder and 1 g NaNO_3_ were added to 80 mL H_2_SO_4_ solution. The mixture was placed in an ice bath with a temperature below 15 °C and was stirred continuously for two hours. A total of 12 g of KMnO_4_ was gradually added during the stirring process. Thereafter, the temperature of the mixture was raised to 35 °C, and the mixture was stirred for another 30 min. Second, the mixture was diluted with 160 mL deionized water and stirred in a room temperature water bath for 15 min. Third, 500 mL deionized water (75 °C) and 30 mL H_2_O_2_ were added to the mixed solution and stirred in a constant-temperature water bath at 75 °C for 4 h. Finally, the mixed solution was pickled and washed until the pH value of the solution was neutral. GO solution was obtained by ultrasonic and centrifugal treatment, and brown GO powder was obtained after freeze-drying for 48 h. SM-GO was synthesized by non-covalent interaction between GO and SMWCNTs. SMWCNTs and GO powders of the same mass were added to deionized water and sonicated for 12 h to prepare a brown solution. SM-GO powder was obtained after freeze-drying for 48 h. The above preparation process is shown in Figure 1.

### 2.3. Preparation of the Coating Samples

First, the 20 carbon steel samples were washed successively with acetone, alcohol, and deionized water. Then, they were polished using 400, 800, and 50 grit silicon carbide waterproof abrasive papers in turn. After polishing, the samples were ultrasonically cleaned in ethanol and water, and dried for reserve. Second, three types of coatings were obtained by adding SMWCNTs, GO, and SM-GO powder (0.3 wt.%) into epoxy zinc-rich coatings and ultrasonically treated for 60 min. Subsequently, the curing agent (10 wt.%) was added to the coating and slowly stirred for 15 min to reduce the formation of bubbles. Finally, the coating was applied to the surface of the carbon steel sample with the brush and cured at room temperature for one week. The thickness of the dry coating was 100 ± 10 μm. In addition, for comparison, the pure epoxy zinc-rich coatings were prepared using the same method.

### 2.4. Characterization Methods

The change of chemical functional groups on the surface of the sample was characterized by Vertex Fourier transform infrared spectrometer. The scan range was 400 to 4000 cm^−1^, and the number of scans was 21. The D8 Advance X-ray diffraction analyzer was used to characterize the molecular structure of the sample, and the lamellar spacing was calculated. Cu target radiation (λ = 0.154 nm) was used, with a tube pressure of 45 kV and tube flow of 50 mA, at a scanning rate of 8 cm/s in the range of 4° to 90°. The Raman spectrum was measured by Horiba Lab-RAM HR Evolution with the focal length of 800 nm, which was used to characterize the molecular structure of the sample.

The OCP and EIS of the coatings were measured using a PARSTAT 2273 electrochemical workstation. A three-electrode system was constructed with a saturated calomel electrode (SCE) as the reference electrode, platinum electrode as the auxiliary electrode, and 3.5 wt.% NaCl as the solution. The frequency range and amplitude voltage of the EIS test were 10 kHz to 0.01 Hz and 10 mV, respectively. The salt spray test of the sample (2 × 25 × 50 mm) was carried out in the salt spray testing chamber, according to the ISO 4628–2005 standard. The surface of the coating sample was forked, and the cut was as deep as the metal substrate. Then, the sample was exposed to 5 wt.% NaCl salt spray. The temperature was kept at 35 °C, and the pH value was 6.5 to 7.2.

The micro-surface morphology of the coating after the salt spray test was observed using the FEI quanta FEG 250 field emission scanning electron microscope (FESEM). Before the test, it was necessary to spray gold on the surface of the sample to ensure electrical conductivity. After 70 days of salt spray test, the coating was peeled off, and the metal substrate was exposed. When the coating was heated at 50 °C for 5 min, the coating became soft. We used tweezers to remove the coating and were careful not to damage the metal substrate. The rust on the metal surface was removed using a descaling solution, and the surface morphology of metal substrate was observed using the German Zeiss Axio Imager A2m 3D confocal microscope.

## 3. Results and Discussion

### 3.1. Characterization Results of GO, SMWCNTs and SM-GO

**Figure 2:**
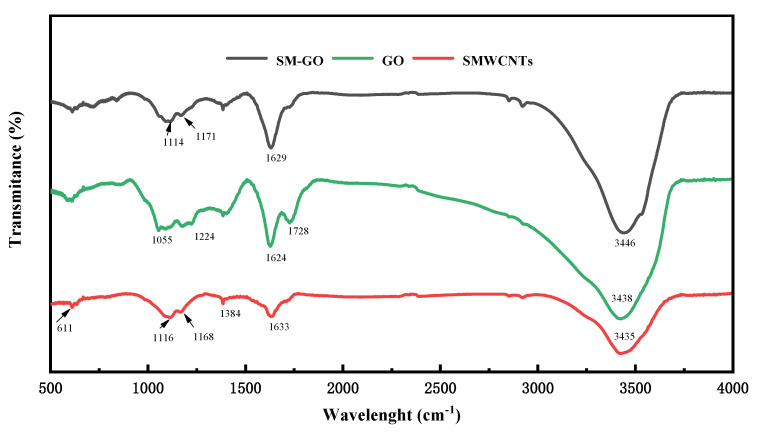
FT−IR characterization of SM−GO, SMWCNTs, GO.

The specific functional groups of the samples were observed from the FT-IR spectrum, reflecting the chemical structure characteristics, as shown in Figure 2. Besides the absorption peaks caused by the unoxidized domain and aromatic ring structure at 1627 cm^−1^ and 611.4 cm^−1^, the GO samples also have absorption peaks at 1055 cm^−1^, 3438 cm^−1^, 1224 cm^−1^, and 1728 cm^−1^, corresponding to the stretching vibrations of the C–O–C bond, O–H bond, C–O bond, and C=O bond, respectively. It shows that there are oxygen-containing groups of the epoxy group, hydroxyl group, and carboxyl group in the sample, which proves that GO is successfully synthesized in this experiment. SMWCNTs also have diffraction peaks caused by the unoxidized domain and aromatic ring structure at 1633 cm^−1^ and 611 cm^−1^. In addition, there are absorption peaks caused by the of the C–O bond and O–H bond at 1384 cm^−1^ and 3435 cm^−1^, and absorption peaks caused by the sulfonic group at 1116 cm^−1^ and 1168 cm^−1^, which proves that MWCNTs are successfully sulfonated. For the SM-GO samples, the absorption peak positions are roughly the same as that of GO. The difference is that the absorption peak at 1114 cm^−1^ becomes a single peak, and the position shifts, which is consistent with that of the sulfonic acid group, proving that the SM-GO is successfully prepared.

**Figure 3:**
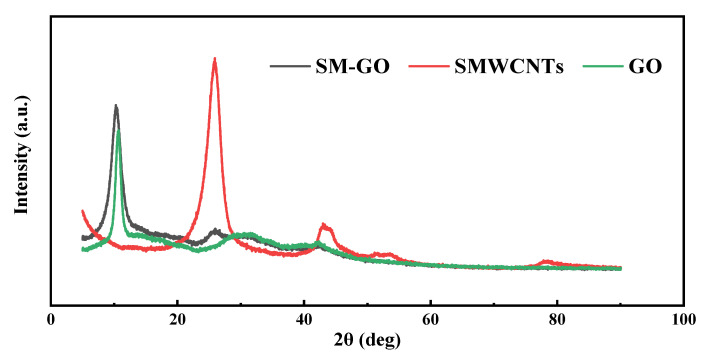
XRD characterization of GO, SMWCNTs and SM-GO samples.

Figure 3 shows the XRD characterization results of GO, SMWCNTs, and SM-GO. Due to the abundant oxygen-containing functional groups, the diffraction peak of GO is located at 2θ = 10.76°. For the SMWCNTs, there are two diffraction peaks at 2θ = 25.96°and 2θ = 42.14°, which belong to the (002) and (100) faces of the hexagonal structure of graphite carbon atom. Compared with the X-ray diffraction card number (JCPDF ICDD5 8-1638) [26], both the diffraction peaks move to a lower 2θ position. This indicates that the interlayer spacing of SMWCNTs increases, which confirms the formation of the required functional groups [27]. Compared with SMWCNTs, the characteristic peak of SM-GO moves to 2θ = 10.25°, which shows that after GO is modified by SMWCNTs, the distribution of its linear structure develops from disorder to order. The interlayer spacing of SM-GO increases, and its dispersion is also improved.

**Figure 4:**
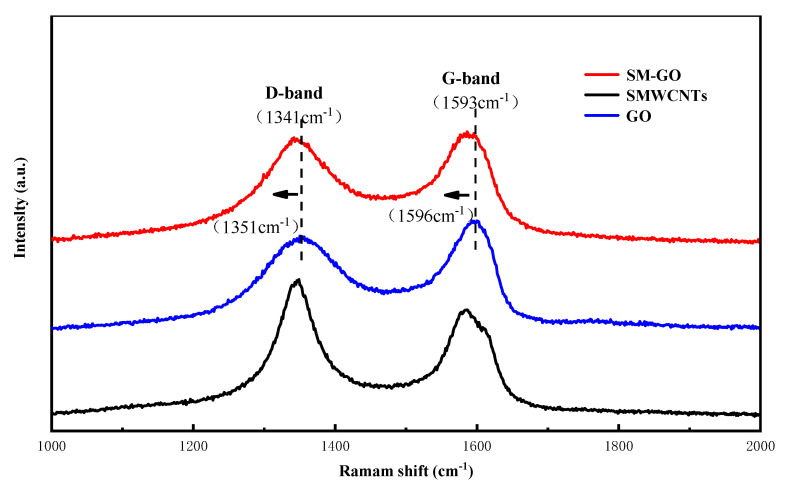
Raman characterization of GO, SMWCNTs and SM−GO samples.

As shown in Figure 4, two leading bands exist in the Raman spectrum. The D- and G-band are related to the breathing mode of A_1g_ symmetry and E_2g_ symmetry of sp^2^ carbon atoms, respectively [3]. After the modification of GO by SMWCNTs, the characteristic peak of the D- (1351 cm^−1^) and G-band (1596 cm^−1^) move to 1341 cm^−1^ and 1593 cm^−1^, respectively, which show that there is charge transfer between SMWCNTs and GO. The strength ratio of the D- to G-band (*I*_D_/*I*_G_) represents the quality of graphitization or defects in the structure of carbon materials. Due to the increase in the number of isolated sp2 domains, the D-band of SM-GO increases, resulting in an increase in *I*_D_/*I*_G_ from 0.85 to 0.95 [28], which proves that GO is successfully modified by SMWCNTs.

### 3.2. Characterization of Corrosion Resistance of Coatings

#### 3.2.1. Open Circuit Potential (OCP)

**Figure 5:**
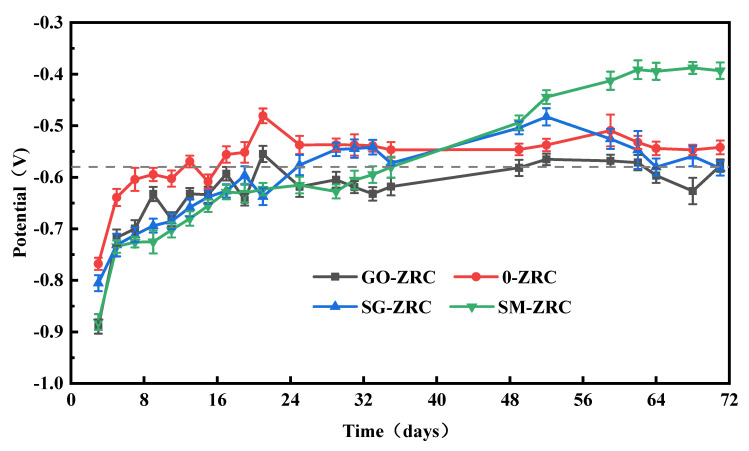
OCP for the different coating samples with immersion time.

Figure 5 shows the variation of OCP values of different coating samples immersed in 3.5 wt.% NaCl solution. The OCP values can reflect the protection characteristics of different coatings [29,30]. When the OCP is lower than the minimum potential value −1.143 V (vs. SCE), the hydrogen evolution occurs. When the OCP is higher than the maximum potential value −0.58 V (vs. SCE), the cathodic protection effect of the coating is invalid [9,31]. In the initial stage of immersion, the OCP values of all samples are less than −0.58 V, indicating that all four coatings have effective cathodic protection. With the increase in soaking time, the OCP value of 0-ZRC samples increases rapidly. The electrolyte solution permeates into the coating matrix through the micropores on the coating surface, which causes the rapid consumption of zinc particles and produces a large number of corrosion products such as ZnO and Zn(OH)_2_. As a result, the electron flow channel between zinc particles is cut off, and the cathodic protection ability of the coating is reduced. After immersion for 16 days, the OCP value of the 0-ZRC sample is higher than that of carbon steel, which indicates that the coating has lost the ability of cathodic protection and entered the shielding stage.

After immersion for 36 days, two coatings containing SMWCNTs (SM-ZRC and SG-ZRC) enter the shielding stage. However, the cathodic protection effect of GO-ZRC is persisting for approximately 50 days. This indicates that the addition of nanoparticles can prolong the cathodic protection time and improve the protective effect of the coating. However, due to the different structure and electrochemical activity of different nanoparticles, the coatings have different utilizations of zinc particles. First of all, the planar structure of GO has a better barrier effect than the tubular structure of SMWCNTs. Secondly, conductive nanoparticles are easy to form a galvanic battery with zinc particles, which triggers galvanic corrosion and accelerates the consumption of zinc particles. The oxygen-containing functional groups on the surface of GO hinder the electron flow. Thus, the electrochemical activity is passivated, and the galvanic corrosion is inhibited. However, SMWCNTs have strong electrochemical activity [25]. When GO is modified by SMWCNTs, the electrochemical activity is also improved. Therefore, SM-ZRC and SG-ZRC samples are prone to severe galvanic corrosion, which decreases the cathodic protection of the coating [32,33]. Hence, the cathodic protection effect of the GO-ZRC sample is the best.

#### 3.2.2. Salt Spray Test

**Figure 6:**
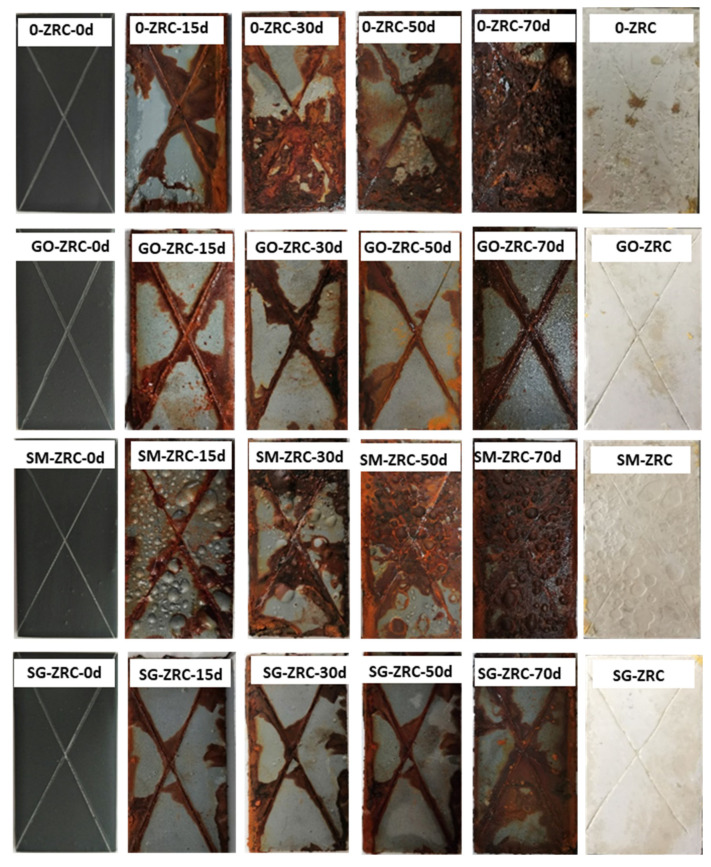
Visual observations of the different coating samples after 0, 15, 30, 50 and 70 d exposure to salt spray; results after peeling of coating after 70 d salt spray.

Figure 6 shows the macroscopic corrosion morphology of each coating after exposure to salt spray for 0 d, 30 d, 30 d, 50 d, and 70 d. In the initial stage of the salt spray test (30 d), rust spots appeared on the bare metal areas (marked lines) on all coating surfaces. Among them, more than a quarter of the 0-ZRC surface was covered with yellow corrosion products, and a small density of blistering phenomenon appeared. The other three sample surfaces were covered with fewer corrosion products, verifying that the 0-ZRC samples were severely corroded in the early stage of the salt spray test. With the penetration of corrosive media and the oxidation of many zinc particles at the metal/coating interface, the corrosion products accumulate and expand at the metal/coating interface, which leads to a further decrease in the corrosion resistance of the coating. With the continuation of the salt spray test, the corrosion degree of 0-ZRC samples is further deepened. After 70 days, the surface of the sample was entirely covered by corrosion products.

After 30 days of the salt spray test, a large area of bubbles appeared on the surface of the SM-ZRC samples, and the bubble size near the exposed area was larger. With the continuation of the salt spray test, the corrosion products accumulated in the bubbles cannot penetrate and release into the environment. To maintain the balance of the internal and external osmotic pressure of the coating bubbles, a large amount of salt spray is absorbed from the outside. The corrosion degree of the coating/metal interface is further aggravated. After 70 days of the salt spray test, the surface corrosion of the SM-ZRC sample was very severe.

GO-ZRC and SG-ZRC samples still had excellent corrosion resistance after 50 days of the salt spray test. This is because the utilization of zinc powder is improved by adding conductive nanoparticles such as GO and SM-GO, and the cathodic protection effect on the exposed metal area is enhanced. In addition, corrosion products and nanoparticles provide proper shielding for the metal matrix and inhibit the expansion and accumulation of corrosive substances at the metal/coating interface. After 70 days, a large number of corrosion products appear on the surface of SG-ZRC, which indicates that the corrosion resistance is greatly inhibited. This is related to the galvanic corrosion caused by SM-GO. However, the surface of GO-ZRC is still covered with fewer corrosion products, and the coating still has an excellent cathodic protection and shielding effect.

**Figure 7:**
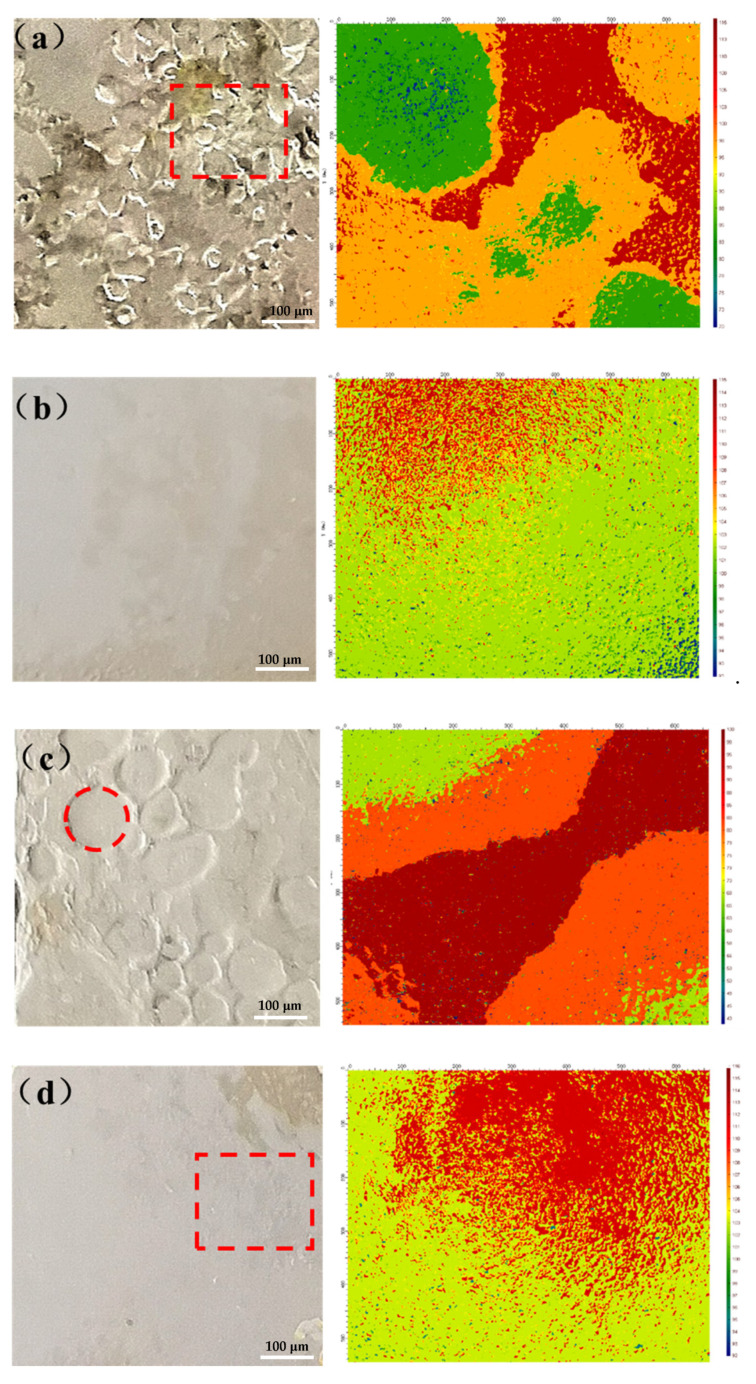
Macroscopic morphology (left) and microscopic 3D morphology (right) of the metal substrate of (**a**) 0-ZRC, (**b**) GO-ZRC, (**c**) SM-ZRC, (**d**) SG-ZRC.

After a salt spray test of 70 days, the coating of the sample was peeled and de-rusted to obtain a bare metal substrate. Dense and irregular corrosion pits on the metal substrate surface were observed with the macroscopic morphology, as shown in Figure 7a. In the microscopic 3D topography of the metal substrate, different colors represent different heights of the metal surface. Red represents the highest position of the metal surface, and blue represents the lowest position of the metal surface. Through the microscopic 3D topography, the rugged surface of the metal substrate of the 0-ZRC sample can be observed. The shape of the corrosion pit was irregular, and the depth was considerable. The maximum height difference was 42.31 μm, which indicates severe corrosion during the salt spray test.

The surface of the metal substrate of the GO-ZRC sample was smooth, and no corrosion pits were observed, as shown in Figure 7b. It is confirmed that GO-ZRC provides sufficient protection for metal substrates. Regular circular etching pits could be observed on the metal substrate surface of SM-ZRC samples, which is related to the galvanic corrosion, as shown in Figure 7c. An evident ladder-like 3D morphology could be observed at the connection of two adjacent corrosion pits. Additionally, the maximum height difference of the metal surface was 54.43 μm. It indicates that SM-ZRC cannot provide enough protection for the metal substrate. Tiny etching pits (square marked) were observed on the metal substrate surface of the SG-ZRC sample, as shown in Figure 7d. The maximum height difference was 16.09 μm, which proves that the metal substrate of the SG-ZRC sample was partially corroded. 

#### 3.2.3. SEM Analysis

**Figure 8:**
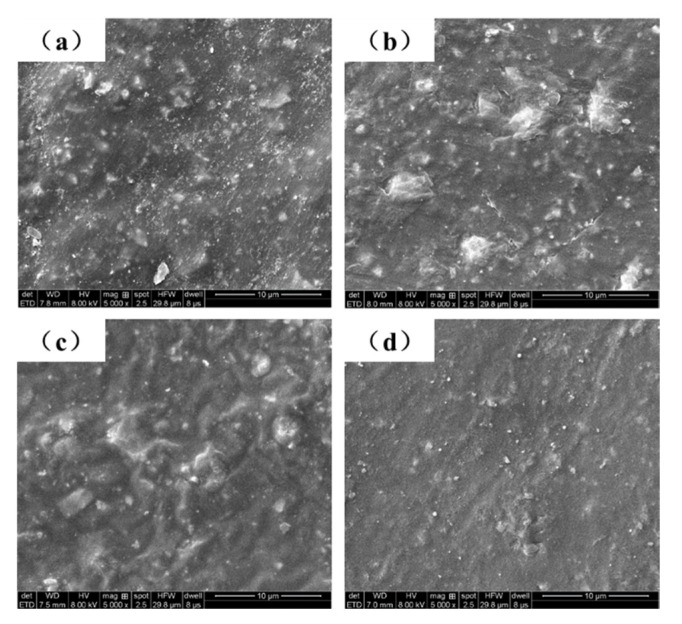
SEM of the 0-ZRC (**a**), GO-ZRC(**b**), SM-ZRC(**c**) and SG-ZRC(**d**) samples.

As shown in Figure 8a, there were micropores on the surface of 0-ZRC samples. When nanoparticles are added to the coating, the porosity of GO-ZRC, SM-ZRC, and SG-ZRC samples decreases, as shown in Figure 8b–d. Simultaneously, the uniform distribution of GO, SMWCNTs, and SM-GO on the surface of the coating can be observed. The dispersion stability of the coating is proved.

**Figure 9:**
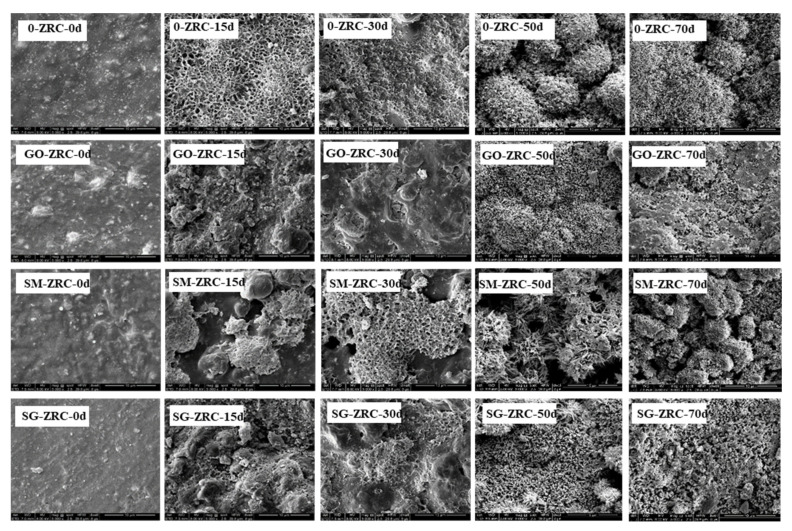
SEM of the 0-ZRC, GO-ZRC, SM-ZRC and SG-ZRC samples after 0, 15, 30, 50 and 70 days exposure to salt spray.

The surface morphology of different coatings after 0, 15, 30, 50, and 70 d exposure to salt spray are shown in Figure 9. After the salt spray test, the surface morphology of the four coatings changes significantly. After 30 days, a large number of corrosion products accumulated on the surface of 0-ZRC. However, only a small amount of corrosion products formed on the surface of GO-ZRC, SM-ZRC, and SG-ZRC samples. This is because the conductive nanoparticles connect a large number of zinc particles into a huge conductive network, and the zinc particles in the entire conductive network are uniformly corroded.

After 30 d of salt spray test, the corrosion degree of the 0-ZRC sample further increased. This is due to the high galvanic corrosion between SMWCNTs and zinc particles. However, the oxidation degree of zinc particles on the surface of GO-ZRC and SG-ZRC samples is still low. After 50 days of salt spray test, loose corrosion products are accumulated on the surface of 0-ZRC and SM-ZRC samples, which increases the corrosion of the metal. However, dense corrosion products are deposited on the surface of SM-ZRC and SG-ZRC samples, which enhance the shielding effect of the coating.

After 70 days of the salt spray test, the corrosion degree of 0-ZRC and SM-ZRC samples further increased. Due to the further accumulation of corrosion products, the original dense structure on the surface of SG-ZRC samples became loose, which caused corrosion of the metal substrate. However, the corrosion products on the surface of GO-ZRC samples still maintained a dense structure, and thus, the coating has a good shielding effect. The SEM results are consistent with the results in Figure 6 and Figure 7.

#### 3.2.4. EIS Analysis

**Figure 10:**
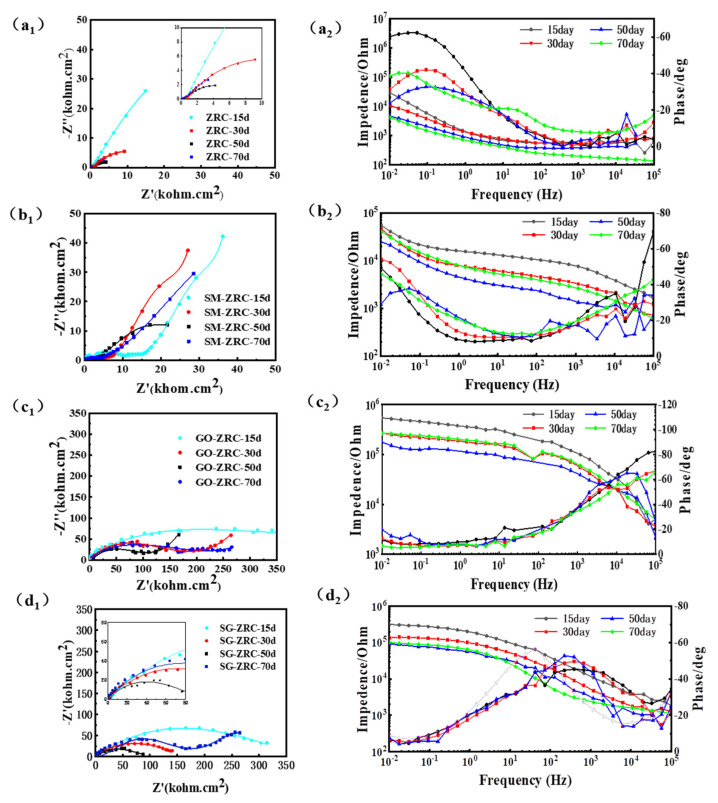
Nyquist and Bode diagrams of the (**a1**,**a2**) 0−ZRC, (**b1**,**b2**) SM− ZRC, (**c1**,**c2**) GO−ZRC and (**d1**,**d2**) SG−ZRC samples after 15, 30,50 and 70 days immersion in 3.5 wt.% NaCl solution.

Figure 10 summarizes the electrochemical evolution behavior of different samples with immersion time in 3.5 wt.% NaCl solution. In general, the capacitive arc radius in the Nyquist diagram can directly reflect the corrosion resistance of the reaction system. It can be concluded that the capacitive arc radius of all coatings decreases in different degrees with the increase of immersion time, which indicates that the corrosion resistance of the coating becomes worse. The capacitive arc radius of GO-ZRC, SM-ZRC, and SG-ZRC is larger than that of 0-ZRC, owing to the addition of conductive nanoparticles to the zinc-rich coating. Furthermore, for different coatings, the shape of the impedance spectrum changes differently with immersion time.

**Figure 11:**
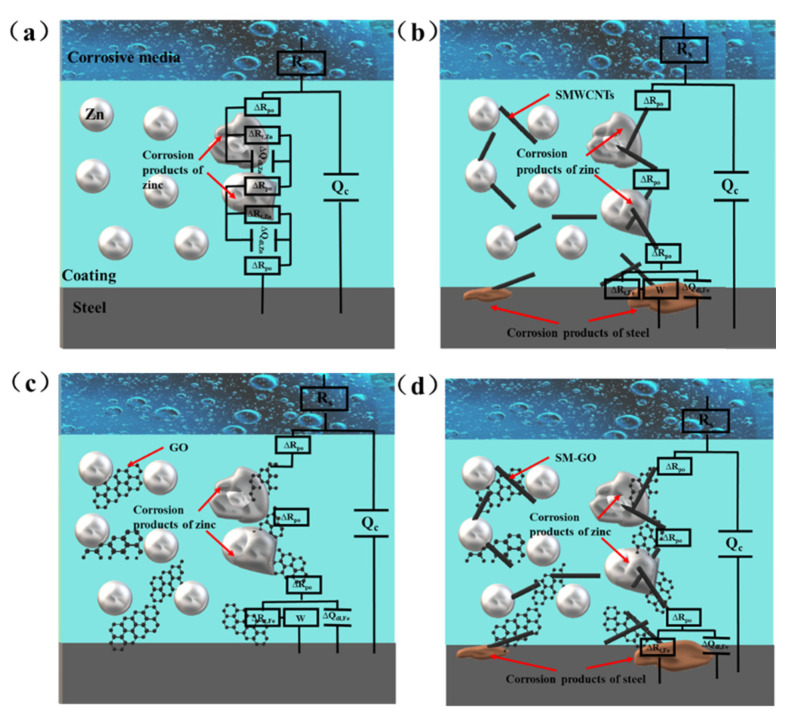
Coating structures and equivalent circuits of (**a**) 0-ZRC; (**b**) SM-ZRC; (**c**) GO-ZRC; (**d**) SG-ZRC.

The EIS diagram of the 0-ZRC sample shows two capacitive reactance arcs, which means there are two time constants, as shown in Figure 10a1. One of the conductive paths contains the coated capacitor C_C_. Due to the dispersion effect, the constant phase angle element Q_C_ is used instead. The same is true in the equivalent circuit below. The other channel includes the ion conduction channel R_po_ in the permeating solution and the electrochemical corrosion process of Zn. The latter includes the electron transfer resistance R_t_ during the corrosion reaction of Zn and the double layer capacitor C_dl_. The equivalent circuit diagram is shown in Figure 11a [2,34,35]. The EIS of the SG-ZRC sample is similar to that of the ZRC sample, and there are two capacitive arcs and two time constants. The Nyquist diagram shows that the impedance arc radius of the SG-ZRC sample becomes smaller in the low frequency band, as shown in Figure 10d1. The equivalent circuits of the two are similar, but their meanings are different. At the same time of the sacrificial reaction of Zn in the sample, serious galvanic corrosion occurred between SM-GO and the metal substrate. Therefore, the equivalent circuit includes an iron-corroded electron transfer resistance R_t,Fe_ and an electric double-layer capacitor Q_dl,Fe_, as shown in Figure 11d. The fitting component parameters for the EIS of 0-ZRC and SG-ZRC in Figure 10 are shown in Table 2.

There is a diffusion characteristic with a slope of 45° in the Nyquist diagram of GO-ZRC samples. The phase angle at the front of the low frequency peak shows an upward trend, as shown in Figure 10c1. Warburg impedance is used in the equivalent circuit to describe the corrosion products of Zn and the diffusion process caused by GO, as shown in Figure 11c. Similar features also exist in the EIS diagram of SM-ZRC samples, as shown in Figure 10b1. On the other hand, there is a process of galvanic corrosion between the SMWCNTs and metal substrate. Therefore, the equivalent circuit of the SM-ZRC sample includes the electron transfer resistance R_t,Fe_ in the iron corrosion reaction, and the diffusion resistance Z_w,Fe_ of the iron corrosion product, as shown in Figure 11b [4,35,36]. The fitting component parameters for the EIS of GO-ZRC and SM-ZRC in Figure 10 are shown in Table 3. There is a positive correlation between the R_po_ value and the physical barrier properties of the coating. The R_t_ value is inversely proportional to the corrosion rate of the metal/coating interface. They are the two basic parameters for evaluating the anti-corrosion performance of the coating. Compared with the parameters in the table, it is found that the R_po_ value of the zinc-rich coating increases significantly after the addition of nanoparticles. This is due to the fact that the nanoparticles fill the micropores of the coating, hindering the penetration of the solution. The barrier property of the coating is related to the dispersion of nanoparticles in the coating. After GO was modified by SMWCNTs, its dispersibility was improved. Therefore, at the initial stage of immersion, the R_po_ value of SG-ZRC samples was as high as 1.93 × 10^5^ Ω·cm^2^. Among all the samples, the barrier performance of SG-ZRC sample was the best. In addition, with the extension of immersion time, it was found that the charge transfer resistance R_t_ of all samples decreased in varying degrees. It means that the corrosion resistance of the coating decreases gradually [37,38,39].

**Figure 12:**
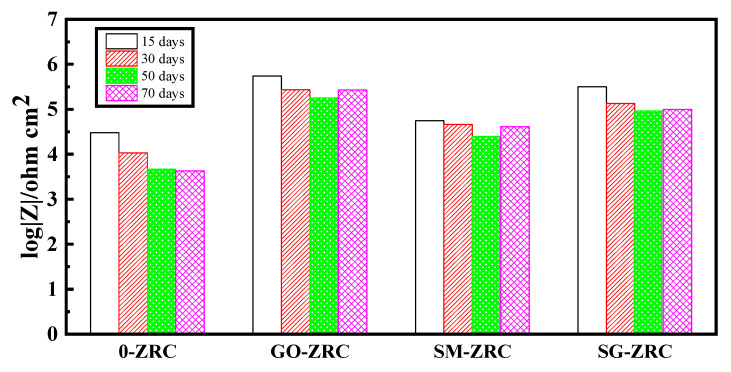
The values of impedance at 10 mHz obtained from Bode diagrams after 15, 30 50, and 70 days immersion in 3.5 wt.% NaCl solution.

The impedance modulus |*Z*| of the lowest frequency (0.01 HZ) in the Bode diagram can be used as an indicator of the impermeability of the coating. The impedance modulus of all samples decreases with the immersion time, which is due to the gradual infiltration of the electrolyte solution into the coating, as shown in Figure 12. After being immersed in the electrolyte solution for 15 days, the impedance modulus of the 0-ZRC sample was 3.0 × 10^4^ Ω·cm^2^, while it is decreased to 1.1 × 10^4^ Ω·cm^2^ and 4.2 × 10^3^ Ω·cm^2^ after soaking for 30 days and 70 days, respectively. The impedance modulus of the samples with nanoparticles is higher than that of 0-ZRC samples, which is due to the barrier properties of the nanoparticles. The impedance modulus of the GO-ZRC sample was 5.5 × 10^5^ Ω·cm^2^, after soaking for 15 days. With the permeation of the electrolyte solution, the impedance modulus decreased to 1.8 × 10^5^ Ω·cm^2^ (soaking for 50 days). Subsequently, the corrosion products form dense protection film on the surface of the metal substrate. The barrier property of the coating is enhanced, and the impedance modulus increased to 2.7 × 10^5^ Ω·cm^2^. After being immersed in the electrolyte solution for 15 days, the impedance modulus of the SM-ZRC sample was 5.5 × 10^4^ Ω·cm^2^, while it is decreased to 2.5 × 10^4^ Ω·cm^2^ after soaking for 50 days. Similar to the case of the GO-ZRC, the impedance modulus of the SM-ZRC increased to 4.1 × 10^4^ Ω·cm^2^ (soaking for 70 days). After soaking for 15 days, the impedance modulus of the SG-ZRC sample was 3.2 × 10^5^ Ω·cm^2^. With the permeation of the electrolyte solution, the impedance modulus decreased to 1.3 × 10^5^ Ω·cm^2^ after soaking for 30 days. After soaking for 70 days, the impedance modulus of the sample decreased to 9.9 × 10^4^ Ω·cm^2^. During the whole immersion process, the impedance modulus of SM-ZRC and SG-ZRC was lower than that of GO-ZRC. This is due to the galvanic corrosion of SM-ZRC and SG-ZRC. The corrosion products destroy the adhesion of the coating to the metal substrate and accelerate the penetration of corrosive substances. Although galvanic corrosion also exists in the SM-GO coating, the barrier performance of SM-GO in the coating is better than that of the SMWCNTs. Therefore, the corrosion resistance of the SG-ZRC sample is greater than that of SM-ZRC. In addition, none of the four coatings swelled or peeled off during the immersion test for 70 days, and the impedance spectrum test results also show that the coating did not enter the failure period, indicating that the coating still protects the substrate metal [6,40,41,42].

## 4. Anti-Corrosion Mechanism

**Figure 13:**
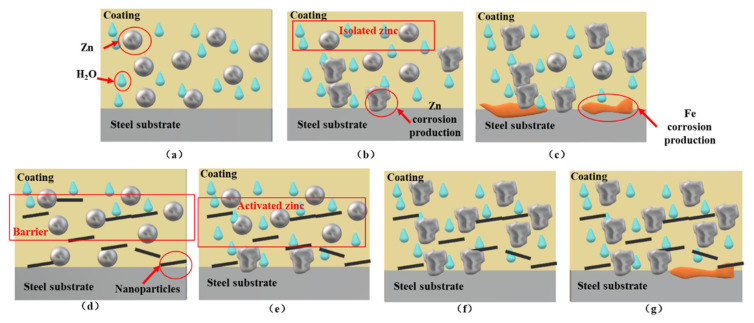
Pure zinc-rich coating (**a**) cathodic protection stage; (**b**) shielding stage; (**c**) failure stage; zinc-rich coating containing nanoparticles (**d**) initial shielding stage; (**e**) cathodic protection stage; (**f**) shielding stage; (**g**) failure stage.

The whole service life of pure epoxy zinc-rich coating can be divided into three stages: cathodic protection stage, shielding stage, and failure stage, as shown in Figure 13a–c, respectively. During the cathodic protection stage, the pores and cracks in the coating matrix provide the penetration path for the corrosive electrolyte solution. Zinc particles are infiltrated by corrosive electrolyte solution and undergo electrochemical corrosion reaction. Some zinc particles (activated zinc) in the coating are electrically connected with the metal substrate and provide cathodic protection for the metal substrate. The remaining zinc particles (isolated zinc) cannot provide cathodic protection for the metal substrate because of the lack of electron flow between the zinc particles and the metal substrate. During the shielding stage, the zinc corrosion products are filled in the penetration path of the corrosive electrolyte solution. It hinders the diffusion of the corrosive electrolyte solution in the coating matrix and protects the metal substrate. In the failure stage, the shielding effect of the coating is lost. A large amount of corrosive media enters the coating, causing serious corrosion of the metal substrate.

Due to the presence of nanoparticles in the three samples (GO-ZRC, SM-ZRC, SG-ZRC), the whole service life can be divided into four stages: initial shielding stage, cathodic protection stage, shielding stage and failure stage, as shown in Figure 13d–g, respectively. In the initial shielding stage, the nanoparticles form many isolation layers in the coating, which prevents the diffusion of corrosive particles such as water and oxygen. In the cathodic protection stage, the conductive nanoparticles become the conductive bridge between the zinc particles to form a conductive network. The proportion of activated zinc is increased, and the utilization rate of zinc particles is improved. Therefore, the cathodic protection time of the coating is prolonged. In the shielding stage, both nanoparticles and corrosion products provide shielding for the metal substrate. During the failure stage, the coating loses protection for the metal substrate. Due to the lack of conductive nanoparticles in the 0-ZRC samples, the cathodic protection and shielding ability are lower than those of the other three samples. Therefore, the corrosion resistance of 0-ZRC is the worst.

The high conductivity of carbon materials such as GO, SMWCNTs, and SM-GO can promote the corrosion of steel and the consumption of zinc particles. However, a large number of oxygen-containing groups on the surface of GO can tremendously reduce the electrical conductivity and electrochemical activity. If the oxygen-containing groups are removed, the reduced GO has a higher positive potential (+0.15 V vs. SCE) than carbon steel (−0.44 V vs. SCE), which will accelerate galvanic corrosion of steel and Zn [43,44]. Therefore, GO can inhibit galvanic corrosion to a certain extent and ensure the corrosion resistance of the coating.

**Figure 14:**
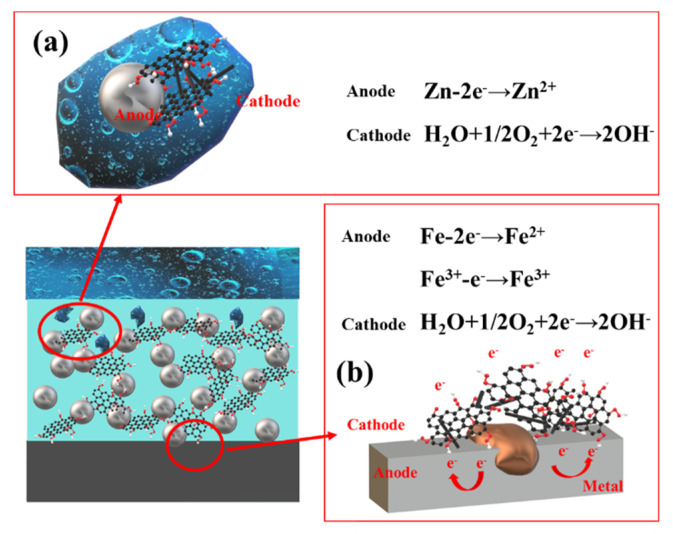
Galvanic corrosion of (**a**) SM−GO and zinc particles; (**b**) SM−GO and metal substrate.

SMWCNTs have strong electrochemical activity and are easy to activate the reaction between cathode and anode. Additionally, SMWCNTs are prone to corrode with zinc particles and metal substrate, as shown in Figure 14. During the longitudinal conduction of the current between the zinc particles and the metal substrate along the SMWCNTs axis, current leakage in the lateral direction (vertical to the SMWCNTs axis) is prone to occur, causing a large proportion of anode current to be wasted. These leakage currents accelerate the cathodic reaction rate and promote the consumption reaction of zinc particles. Due to the existence of GO, the galvanic corrosion of SM-GO is weaker than that of SMWCNTs. However, the electrochemically active SM-GO acts as the cathode, and the metal substrate acts as the anode. Galvanic corrosion occurs between them, resulting in a decrease in the corrosion resistance of the coating. In addition, the cathode SM-GO has a high specific surface area, and it is easy to form a “large cathode, small anode” with the anode zinc or metal substrate, which will accelerate corrosion [13,45,46]. Therefore, the corrosion resistance of the SG-ZRC sample is better than that of the SM-ZRC sample, but worse than that of the GO-ZRC sample.

## 5. Conclusions

Through the above studies, the main conclusions are drawn below.

(1) GO is successfully non-covalently modified by SMWCNTs to form SM-GO, of which the interlayer spacing increases, and the dispersion in the coating matrix is improved.
(2) Due to the conductivity and physical shielding of the nanoparticles, the zinc-rich coatings with conductive nanoparticles have a longer cathodic protection time and a larger impedance modulus than pure zinc-rich coatings. That is, the corrosion resistance of the composite coating is enhanced.
(3) SMWCNTs have strong galvanic corrosion with zinc particles and metal substrates. Due to the presence of oxygen-containing functional groups, the galvanic corrosion of GO is relatively weak. Thus, the galvanic corrosion effect of SM-GO lies between them. Therefore, the regularity of the corrosion resistance of the coatings is recorded as GO-ZRC > SG-ZRC > SM-ZRC.
(4) It is proved that carbon nanotubes should not be used to modify GO in zinc-rich coatings. When modifying GO, attention should be paid to eliminating its cathodic behavior in order to reduce the galvanic corrosion.

## Figures and Tables

**Table 1:** Summary of the modification methods of graphene.

Additives	Coating	Descriptions	Ref.
Polyaniline/ graphene oxide (GO-PANI)	Epoxy zinc-rich coating	Both the cathodic protection properties and barrier performance of the ZRC are improved after addition of 0.1 wt.% GO and GO-PANI nanosheets to the ZRC sample.	[9]
Fluorinated reduced graphene oxide (FrGO-IL)	Waterborne epoxy coating	It improves the corrosion resistance performance of the coating by exerting the superior shielding effect and inhibiting the ability for micro-galvanic corrosion.	[17]
Silanized graphene oxide (SGO)	Silane coating	The composite coatings exhibit the highest protective properties with the addition of 0.2 wt.% of SGO.	[18]
Polydopamine-coated graphene oxide (GO-PDA)	water-borne epoxy coating	The results demonstrate that the inclusion of well-dispersed GO-PDA nanosheets leads to a remarkable improvement in the corrosion protection performance of water-borne EP coating.	[19]
Graphene oxide/multi-walled carbon nanotubes (GO-MWCNTs)	Urushiol formaldehyde polymer coating	The resulting GO/MWCNTs/UFP films exhibit outstanding properties in terms of electrochemical corrosion, alkali-resistance, hardness and adhesion.	[20]
Acidified multi-walled carbon nanotubes and gallic acid modified graphene oxide flakes (FMWCNT-FGO)	Polyurethane coating	The composite coatings simultaneously underwent no corrosion after immersion for 96 h, and the corrosion resistance was better than that of the other coatings.	[21]

**Table 2:** The impedance parameters of 0-ZRC and SG-ZRC.

	Coating	0-ZRC-15d	0-ZRC-30 d	0-ZRC-50 d	0-ZRC-70 d	SG-ZRC-15 d	SG-ZRC-30 d	SG-ZRC-50 d	SG-ZRC-70 d
Parameter									
*R*_s_ (Ω·cm^2^)		5.58 × 10^3^	5.37 × 10^3^	3.75 × 10^3^	1.53 × 10^3^	1.63 × 10^3^	1.13 × 10^3^	0.97 × 10^3^	1.36 × 10^3^
*Y*_QC_ (Ω^−1^·cm^−2^·s^n^)		2.51 × 10^−4^	6.95 × 10^−5^	8.07 × 10^−5^	8.09 × 10^−4^	5.98 × 10^−7^	6.39 × 10^−7^	5.53 × 10^−7^	2.19 × 10^−6^
n_QC_		0.716	0.812	0.961	0.387	0.573	0.661	0.753	0.679
*R*_po_ (Ω·cm^2^)		1.18 × 10^4^	4.55 × 10^3^	2.41 × 10^2^	4.45 × 10^2^	1.93 × 10^5^	6.47 × 10^4^	2.48 × 10^4^	8.80 × 10^3^
*R*_t,Zn_ (Ω·cm^2^)		1.29 × 10^4^	1.91 × 10^4^	8.22 × 10^3^	4.45 × 10^3^	-	-	-	-
*Y*_dl,Zn_ (Ω^−1^·cm^−2^·s^n^)		2.78 × 10^−4^	3.37 × 10^−4^	6.52 × 10^−4^	8.09 × 10^−4^	-	-	-	-
n_dl,Zn_		0.747	0.644	0.543	0.397	-	-	-	-
*R*_t,Fe_ (Ω·cm^2^)		-	-	-	-	1.30 × 10^5^	8.18 × 10^4^	6.70 × 10^4^	5.40 × 10^3^
*Y*_dl,Fe_ (Ω^−1^·cm^−2^·s^n^)		-	-	-	-	3.53 × 10^−6^	4.04 × 10^−6^	7.62 × 10^−6^	4.80 × 10^−6^
n_dl,Fe_		-	-	-	-	0.535	0.513	0.459	0.789
Chi-Square		1.3 × 10^−4^	3.1 × 10^−4^	2.0 × 10^−4^	2.3 × 10^−4^	1.4 × 10^−4^	5.7 × 10^−4^	2.8 × 10^−4^	3.0 × 10^−4^

**Table 3:** The impedance parameters of GO-ZRC and SM-ZRC.

	Coating	GO-ZRC-15 d	GO-ZRC-30 d	GO-ZRC-50 d	GO-ZRC-70 d	SM-ZRC-15 d	SM-ZRC-30 d	SM-ZRC-50 d	SM-ZRC-70 d
Parameter									
*R*_s_ (Ω·cm^2^)		1.00 × 10^−7^	6.63 × 10^−7^	1.20 × 10^−4^	7.50 × 10^−3^	1.24 × 10^−5^	6.52 × 10^−3^	1.00 × 10^−7^	1.00 × 10^−3^
*Y*_QC_ (Ω^−1^·cm^−2^·s^n^)		2.19 × 10^−6^	1.88 × 10^−8^	2.76 × 10^−8^	4.32 × 10^−6^	9.82 × 10^−7^	3.30 × 10^−22^	5.05 × 10^−5^	5.96 × 10^−6^
n_QC_		0.072	0.721	1.000	0.062	0.477	0.038	0.226	0.403
*R*_po_ (Ω·cm^2^)		1.00 × 10^5^	9.60 × 10^4^	5.04 × 10^4^	6.19 × 10^4^	1.39 × 10^4^	2.41 × 10^3^	4.35 × 10^3^	1 × 10^2^
*R*_t,Zn_ (Ω·cm^2^)		5.53 × 10^5^	1.29 × 10^4^	3.35 × 10^4^	9.34 × 10^3^	-	-	-	-
*Y*_dl,Zn_ (Ω^−1^·cm^−2^·s^n^)		3.83 × 10^−10^	1.79 × 10^−6^	1.25 × 10^−6^	2.30 × 10^−9^	-	-	-	-
n_dl,Zn_		1.000	0.401	0.586	0.843	-	-	-	-
Z_W,Zn_		7.17 × 10^−8^	4.91 × 10^−5^	5.34 × 10^−5^	8.67 × 10^−8^	-	-	-	-
*R*_t,Fe_ (Ω·cm^2^)		-	-	-	-	9.95 × 10^4^	4.97 × 10^3^	1.10 × 10^3^	5.30 × 10^3^
*Y*_dl,Fe_ (Ω^−1^·cm^−2^·s^n^)		-	-	-	-	1.23 × 10^−4^	1.58 × 10^−6^	9.74 × 10^−5^	5.87 × 10^−10^
n_dl,Fe_		-	-	-	-	0.635	0.515	0.638	1.000
Z_W,Fe_(Ω^−1^·s^−0.5^)		-	-	-	-	1.79 × 10^−10^	1.12 × 10^−4^	2.30 × 10^−4^	1.03 × 10^−4^
Chi-Square		7.0 × 10^−4^	2.1 × 10^−4^	3.5 × 10^−4^	6.4 × 10^−4^	3.2 × 10^−4^	3.7 × 10^−4^	5.4 × 10^−4^	4.4 × 10^−4^

## Data Availability

The raw/processed data required to reproduce these findings cannot be shared at this time due to technical or time limitations.
